# A Graded Exposure, Locomotion-Enabled Virtual Reality App During Walking and Reaching for Individuals With Chronic Low Back Pain: Cohort Gaming Design

**DOI:** 10.2196/17799

**Published:** 2020-08-10

**Authors:** Rebecca White Hennessy, Deanna Rumble, Mike Christian, David A Brown, Zina Trost

**Affiliations:** 1 PhD Program in Rehabilitation Science School of Health Professions University of Alabama at Birmingham Birmingham, AL United States; 2 Department of Psychology University of Alabama at Birmingham Birmingham, AL United States; 3 From The Future LLC Denton, TX United States; 4 School of Health Professions University of Texas Medical Branch Galveston, TX United States; 5 ​Department of Physical Medicine and Rehabilitation Virginia Commonwealth University Richmond, VA United States

**Keywords:** virtual reality, chronic low back pain, walking, rehabilitation, virtual reality exposure therapy

## Abstract

**Background:**

Chronic low back pain (cLBP) can interfere with daily activities, and individuals with elevated pain-related fear (also known as kinesiophobia or the fear of injury due to movement) can develop worse long-term disability. Graded exposure (GEXP) protocols use successive participation in avoided activities to help individuals overcome fearful movement appraisals and encourage activity. We sought to develop a series of GEXP virtual reality (VR) walking and reaching scenarios to increase the exposure and engagement of people with high kinesiophobia and cLBP.

**Objective:**

This study aims to (1) determine GEXP content validity of the VR application and (2) determine the feasibility of individuals with cLBP performing locomotion-enabled physical activities.

**Methods:**

We recruited 13 individuals with cLBP and high pain-related fear to experience six VR modules, which provide progressive movement exposure over three sessions in a 1 week period. At session 1, participants ranked each module by likelihood to avoid and assigned an expected pain and concern for harming their back rating to each module. Participants provided a rating of perceived exertion (RPE) after experiencing each module. To test feasibility, we administered the system usability scale (SUS) and treatment evaluation inventory (TEI) following the final session. In addition, we measured pain and pain-related fear at baseline and follow-up.

**Results:**

The 12 participants who completed the study period assigned higher avoidance (*P*=.002), expected pain (*P*=.002), and expected concern (*P*=.002) for session 3 modules compared with session 1 modules. RPE significantly increased from session 1 (mean 14.8, SD 2.3) to session 3 (mean 16.8, SD 2.2; *P*=.009). The VR application showed positive feasibility for individuals with cLBP through acceptable SUS (mean 76.7, SD 13.0) and TEI (mean 32.5, SD 4.9) scores. Neither pain (*P*=.20) nor pain-related fear (*P*=.58) changed significantly across sessions.

**Conclusions:**

The GEXP VR modules provided progressive exposure to physical challenges, and participants found the VR application acceptable and usable as a potential treatment option. Furthermore, the lack of significant change for pain and pain-related fear reflects that participants were able to complete the modules safely.

## Introduction

### Background

Chronic low back pain (cLBP)—low back pain present for longer than 3 months—is a common symptom with an estimated lifetime prevalence of 80% and is the second leading cause of disability in the United States [1,2]. Treatment guidelines include implementing self-care strategies (remain active, apply superficial heat, etc) and pharmacological (acetaminophen, nonsteroidal anti-inflammatory drugs, etc) and nonpharmacological treatments (exercise therapy, cognitive behavioral therapy, etc), but many cases resist traditional treatment [3]. Historically, the biomedical model of pain described the experience of pain solely through biological mechanisms and suggested a predictable and linear relationship between pain and tissue damage. However, this straightforward relationship has failed to explain many clinical observations of pain and has led to the uptake of a more biopsychosocial approach to chronic pain, which considers the experience of pain as a dynamic interaction between biological, sociocultural, and psychological factors [4,5].

The fear-avoidance model (FAM), a widely used theory that attempts to explain the development of chronic disability after a back injury, identifies pain-related fear as a central cognition that predicts long-term disability after musculoskeletal injury [6-9]. Elevated pain-related fear is the belief that pain always signals serious tissue damage, and the FAM postulates that individuals with high pain-related fear will avoid physical activities that they believe will further exacerbate pain, trigger reinjury, or prevent recovery. Elevated pain-related fear greatly affects movement quality and is related to slower walking speeds [10], modified reaching strategies [11], and reduced lifting ability [6]. In line with the FAM, an estimated 7.6 million Americans report disability related to back problems, including cLBP [2]. Accordingly, interventions that aim to increase physical function and decrease disability may have greater personal and societal impact than interventions that solely focus on decreasing pain intensity [12-14].

### Graded Exposure as an Approach to Alleviate Pain-Related Fear

Treatments based on the FAM employ strategies to reduce pain-related fear and activity avoidance. Graded exposure (GEXP) is one such treatment, in which individuals with cLBP and high pain-related fear rank activities based on fearfulness and then progressively confront these fearful appraisals through activity [15]. The photographic series of daily activities (PHODA) is a measurement tool commonly used to capture how individuals rank expected pain and the harm of different daily activities [16,17]. The PHODA includes activities that participants can rank by perceived harm, such as walking while carrying shopping bags, twisting to take books off a shelf, and mowing the lawn.

GEXP protocols aim to correct catastrophic misinterpretations of pain sensations and reduce expectations of harm to the back, leading to functional improvements. A small number of randomized controlled trials have demonstrated that GEXP may clinically reduce fear of movement and disability among cLBP patients [18,19]. However, low patient engagement, high dropout, and limited ability to provide exposure to specific activities may limit the success of GEXP. Additionally, GEXP in a clinical setting relies on the availability of physical space and quality of clinical props to model relevant and lifelike exposure scenarios, which often results in training scenarios that are uninspiring and irrelevant to patient goals [20].

### Virtual Reality as a Therapeutic Tool to Deliver GEXP

Virtual reality (VR) is a tool that can generate environments not otherwise possible in a clinical or laboratory setting. Previous research suggests that virtual environments can enhance rehabilitative training and improve physical outcomes [21-24]. GEXP VR protocols may enable clinicians to prescribe training scenarios that are not feasible in a traditional clinical setting. Specifically, VR may enhance traditional GEXP by offering tailored training based on patient goals, reduced clinician workload, and improved patient monitoring through movement tracking [25]. VR can generate an unlimited number of objects and environments that may enhance patient interaction and improve the intrinsic motivation of GEXP therapy.

Researchers and clinicians can categorize movement tasks by body orientation (providing stability or transport), environment (stationary or in motion), and object manipulation requirements (holding an object or not) [26,27]. Several VR applications have already shown promising feasibility and usability in individuals with cLBP [28,29], but to date, they have only provided exposure to back-challenging activities (ie, reaching) from a stationary position. Stationary reaching tasks can include object manipulation (eg, reaching with an object) and a dynamic environment, but they limit users to reaching from a seated or standing position. Although this may be appropriate for some users, this approach neglects reaching activities that are more relevant to daily living that require body transport. Thus, these systems limit users and force training to stop before more complex tasks, such as movement that requires walking.

In response, we have developed an engaging, locomotion-enabled GEXP VR application to address the lack of applications that provide progressive movement challenges for individuals with cLBP. The VR application, Lucid, consists of six 3-min modules that challenge participants to complete engaging activities in VR that require progressively more challenging walking and reaching movements in real life. The progressive modules deliver exposure over 3 study sessions in a weeklong training period, where users experience 2 modules at each session.

### Objectives

Before evaluating the efficacy of the GEXP VR application on pain-related health outcomes, we needed to evaluate the basic parameters of the application’s GEXP content and establish how potential users respond to the application. In this study, we aimed to measure the *GEXP content* (ie, How well did the module goals gradually challenge participants with fear-inducing movements) and *feasibility* (ie, Is it possible for participants with cLBP to use the app?). To measure feasibility, we specifically examined *usability* (ie, Can users accomplish module goals with effectiveness, efficiency, and satisfaction? [30]), *safety* (ie, Can users complete the module tasks without experiencing harm?), and *acceptance* (ie, Do users find the potential treatment option fair, reasonable, and appropriate? [31]).

#### Hypothesis 1: GEXP Content

Before exposure to the VR modules, participants would report higher expected ranked avoidance, higher expected pain ratings, and higher expected back-related concern ratings for the more challenging modules compared with less challenging modules.

#### Hypothesis 2: Acceptability and Usability

Participants would report that the VR application is acceptable and usable, as determined by acceptability and usability scores above the respective cutoff values.

#### Hypothesis 3: Safety

The VR application would be safe for participants with cLBP to complete, as determined by no significant increase in pain or pain-related fear over the study period.

## Methods

### Equipment

**Figure 1:**
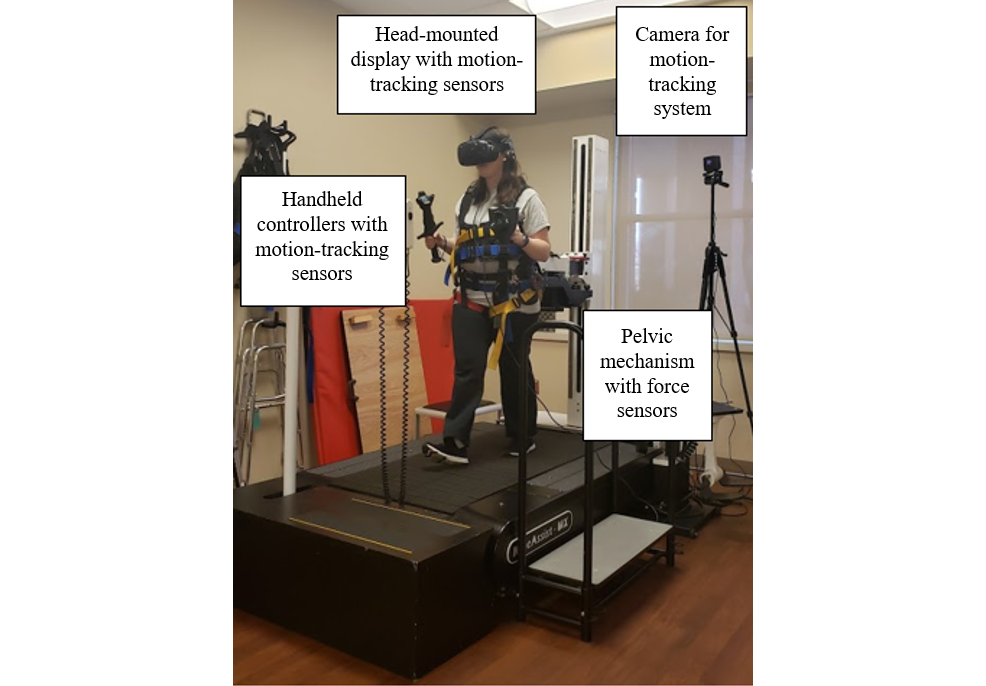
Walking and reaching virtual reality application setup.

**Figure 2:**
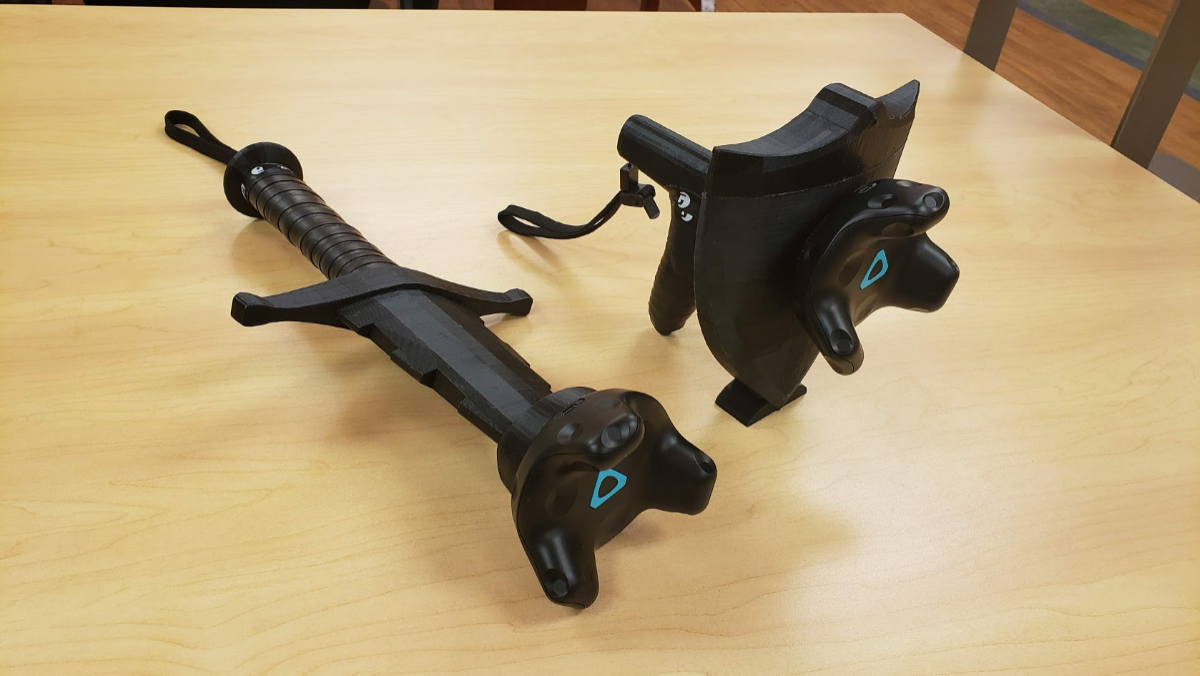
Weighted sword and shield hand-held controllers.

We used a commercially available self-driven treadmill and a VR system to deliver the VR walking experience (Figure 1). Participants wore an HTC-Vive head-mounted display (HMD). Depending on the module, participants held either a regular HTC-Vive controller in each hand or a heavier, custom-made controller weighing approximately 2 lbs (Figure 2). The KineAssist-MX (KA-MX), a specialized self-driven treadmill, allowed participants to set their own walking pace in real time and naturally translate in the VR environment [32,33]. The KA-MX provides a safe walking environment, and several other studies involving both nonimpaired and clinical populations have used it [34-37]. The KA-MX consists of a pelvic mechanism that attaches to participants via a pelvic harness and allows participants normal hip range of motion [38]. As a participant intends to take a step, the pelvic mechanism detects the force generated by the participant’s movement, and a built-in algorithm converts this force to a proportional speed on the treadmill belt. Participants could speed up, speed down, or change direction simply by moving with the intended force in the intended direction. We added a 2-kg upward force through the device’s unweighing function to counteract the weight of the pelvic mechanism on the participants. The KA-MX sent the treadmill speed to a computer via an ethernet cable, and the HTC-Vive system tracked head and hand position. Users walked, reached, and crouched in real life to move around in the VR environment.

### VR Development

We designed and developed the novel VR application, Lucid, that encourages participants to practice real-world movement tasks in a fun and engaging VR world. First, we identified functional movements used in real-world activities by using the movements included in the PHODA. The PHODA includes 8 movement types (ie, lifting, bending, turning, reaching, falling, intermittent load, unexpected movement, long-lasting load instance, or sit with limited dynamics) performed in either static or dynamic positions [39]. From these movements, we selected reaching, bending, and long-lasting loads to incorporate into the VR walking challenges because they have been used in previous studies to provide a graded challenge for individuals with cLBP [16,40]. Second, we designed VR activities that required different levels of each movement task to complete. These VR activities included goals such as fighting monsters, crouching under branches, saving animals that challenged participants to reach, bend to get under obstacles, and walk quickly. We then incorporated the VR activities into six 3-min modules that encouraged participants to complete progressively more difficult combinations of the movement tasks (Table 1). We created the VR application, named Lucid, specifically for this study through a Small Business Innovation Research grant partnership with From the Future, LLC. We used Unity to develop the VR application, which ran on a Windows 10–based personal computer.

**Table 1:** Movement requirements and activity goals for the virtual reality modules.

Session	Module	Movement requirements	VR^a^ activity goal
1 (low-intensity challenge)	1	Walking: any pace Reaching: requires one hand Bending: no Carry weights: no	“Walk at your own pace and rid the realm of monsters. Swing your sword to damage foes and block their attacks with your shield.”
1 (low-intensity challenge)	2	Walking: walking quickly Reaching: requires one hand Bending: no Carry weights: no	“Walk at an increased pace to save as many animals as you can. Monsters have started to prey on the wildlife, and it’s up to you to save the animals before the monster consumes them.”
2 (medium-intensity challenge)	3	Walking: any pace Reaching: requires both hands Bending: no Carry weights: no	“The monsters have desolated the land, and it’s up to you to collect food and coins for the realm. You are given two swords to reach both your foes and your items in all directions.”
2 (medium-intensity challenge)	4	Walking: any pace Reaching: requires one hand Bending: yes Carry weights: no	“Crouch under trees and tunnels to explore more of the realm. You’ll want to make sure you avoid limbs and the ceiling, or you’ll bring your journey to an end.”
3 (high-intensity challenge)	5	Walking: any pace Reaching: requires both hands Bending: yes Carry weights: yes	“Wield a weighted sword and shield while you crouch under trees and tunnels to explore more of the realm. You’ll want to make sure you avoid limbs and the ceiling, or you’ll bring your journey to an end.”
3 (high-intensity challenge)	6	Walking: walking quickly Reaching: requires both hands Bending: yes Carry weights: yes	“Wield a weighted sword and shield to defeat your enemies.”

^a^VR: virtual reality.

**Figure 3:**
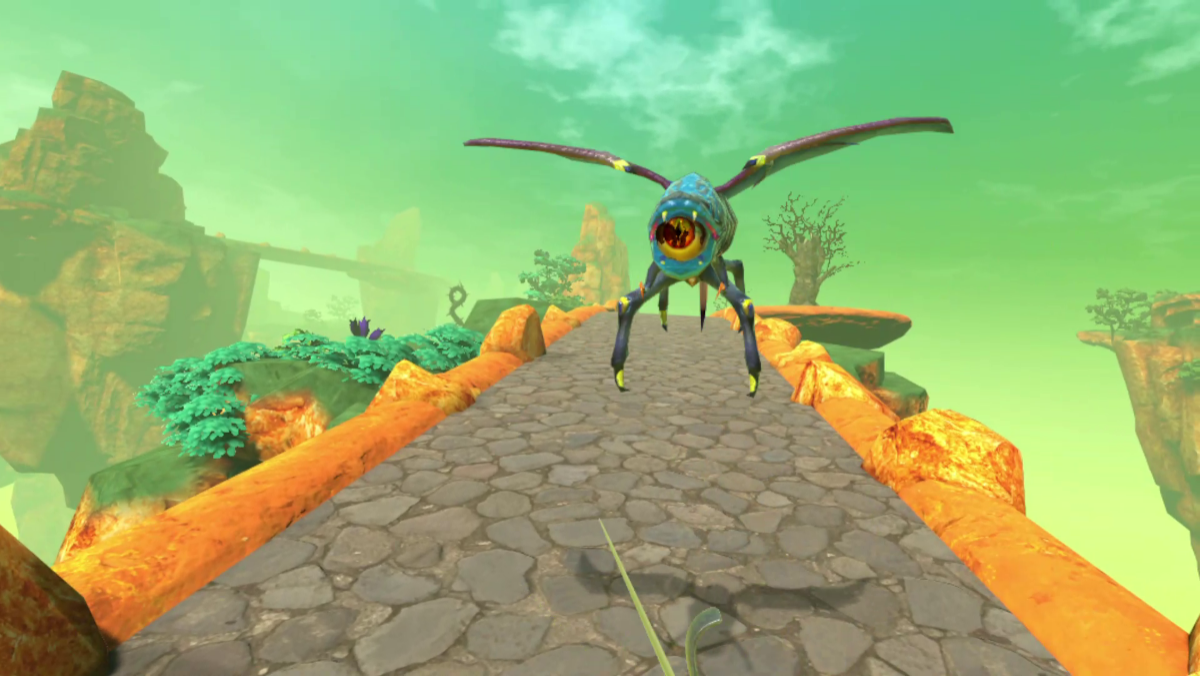
View through the head-mounted display as a user combats a monster while holding the virtual reality sword and shield.

In the HMD, participants navigated a walking path and utilized a VR sword and shield (Figure 3). Participants walked to translate along the path and moved the HTC-Vive controllers to wield the sword and shield. The VR application presented module challenges throughout the trail. Participants did not have to reach a certain distance in the trail or score a certain number of points, so not accomplishing the goal because of walking speed, reaching ability, or bending ability was not a possible outcome. Rather, we simply instructed the participants to do their best and focus on the module objective for the entire 3 min. Additionally, certain in-game collectible objects temporarily equipped the participants with a special ability (eg, longer sword) to help them with their goal.

### Participants

We recruited potential participants through fliers, through the web-based clinical trial registration, and from individuals who participated in previous studies in our lab. We included potential participants aged 18-65 years so that we could test the feasibility in a wide age range of individuals who may benefit from a GEXP intervention. We screened individuals over the phone, and individuals were deemed eligible if they self-reported low back pain for longer than 3 months, experienced interference caused by their back pain in daily life, and reported elevated pain-related fear. Participants were determined to have a high level of fear if they scored greater than 10 points on a 4-question pain-related fear screen that consisted of the 4 highest loaded items from the Tampa scale for kinesiophobia (TSK) [41]. Exclusion criteria included not passing a medical screen (eg, inability to stand for 15 min, recent fall), pregnancy, and significant medical conditions that impair movement ability (eg, arthritis and plantar fasciitis). Additionally, we excluded participants involved in an active training study or a legal claim so that our protocol did not influence other proceedings where pain status is an important outcome. Author DB, a physical therapist, interviewed each participant at the beginning of the study period to determine that each participant had low back pain in a chronic state and did not have a secondary condition that would interfere with participation.

There were 12 participants (aged 43-60 years) with cLBP and high pain-related fear that completed the study protocol (Table 2). One additional participant enrolled in the study but was unable to schedule the VR sessions within 1 week, and we excluded this participant from the analysis. Participants reported an average pain of 6.4 (SD 2.1) for the previous 7 days before the first study session on a numeric rating scale (0 for no pain to 10 for worst imaginable pain). In total, 58% (7/12) of participants were taking pain medications, none of the participants had undergone surgery, and none of the participants were in a physical therapy program at the time of the study. Most participants (8/12, 67%) had back pain for more than 5 years.

**Table 2:** Participant characteristics (N=12).

Characteristics		Values
Age (years), mean (SD)		54.3 (5.1)
**Gender, n (%)**		
	Male	4 (33)
	Female	8 (67)
**Race, n (%)**		
	Black	12 (100)
MPQ-sf^a^ PRI^b^ (possible score range 0-45), mean (SD)		14.8 (9.4)
MPQ-sf VAS^c^ (possible score range 0-100), mean (SD)		52.3 (23.6)
**MPQ-sf PPI** ^d^ **, n (%)**		
	No pain	0 (0)
	Mild	1 (8)
	Discomforting	0 (0)
	Distressing	10 (83)
	Horrible	1 (8)
	Excruciating	0 (0)
TSK^e^ (possible score range 17-68), mean (SD)		44.2 (8.0)
Walking speed in VR^f^ (m/s), mean (SD)		1.1 (0.2)

^a^MPQ-sf: McGill pain questionnaire-short form.

^b^PRI: pain rating index.

^c^VAS: visual analog scale.

^d^PPI: present pain intensity.

^e^TSK: Tampa scale for kinesiophobia (scores >37 are indicative of high fear).

^f^VR: virtual reality.

### Study Design

Participants attended 3 VR sessions (sessions 1-3) over a 1-week period and a follow-up session 3 to 5 days after session 3. During each VR session, participants tested 2 Lucid VR modules in progressive order. Participants completed a baseline questionnaire at the beginning of session 1 and then a follow-up questionnaire at the follow-up session. The questionnaire at session 1 included basic demographics, including gender, age, and race. The institutional review board at the University of Alabama at Birmingham approved the study design, all participants provided written informed consent, and we compensated participants for their time. The study sessions were conducted at a laboratory in the University of Alabama at Birmingham.

### Primary Measures of GEXP Content Validity (Hypothesis 1)

Our primary *GEXP content validity* outcome was the avoidance rank, expected pain, and expected concern participants assigned to each Lucid module at baseline. Developed in the same style as the PHODA, we developed a card for each module that showed an avatar performing the movement required in the module. We laid the 6 cards randomly in front of each participant. Participants organized the cards in a row from the activities they would be least to most likely to avoid. We discreetly recorded the card’s position as the *ranked avoidance* (1 for least likely to avoid to 6 for most likely to avoid), which we used as a measure of how participants perceived the difficulty of the modules in relation to the other modules. Then, to encourage participants to more critically evaluate the tasks in each module, participants reported their *expected pain* if they were to perform the activity (0 for no pain to 100 for worst possible pain) and their *expected concern and worry for harming their back* (0 for not at all concerned to 10 for extremely concerned) for each card.

### Secondary Measures of GEXP Content Validity

Secondarily, we also asked participants to provide their rating of perceived exertion (RPE) after each VR module to measure how participants rated their perceived effort during each VR module [42]. To administer the RPE, we asked participants, “How hard were you working during that activity?” after each module. The RPE scale ranges from 6 (no exertion) to 20 (maximal exertion), and we expected participants to assign higher exertion for the modules with higher challenge intensity. RPE is an accurate measure of perceived effort in individuals with cLBP [43]. Additionally, at session 3, we asked participants to rate the difficulty of the session modules compared with the previously experienced modules (questions included in Multimedia Appendix 1). For each question, participants could respond from 0 (much easier) to 10 (much more difficult).

### Primary Measures of Feasibility (Hypotheses 2 and 3)

At the follow-up session, we administered the treatment evaluation inventory-short form (TEI-sf) as the primary measure of *acceptability*. The TEI-sf is a 9-item questionnaire used to measure intervention acceptability from the participants’ point of view. Scores range from 9 to 45, and scores above 27 are considered acceptable [31]. The TEI-sf is a valid measure [31], and several other studies have used it to measure acceptability in participants with back pain [44,45]. We also administered the system usability scale (SUS) at follow-up as our primary measure of *usability*. The SUS is a reliable 10-item usability measure and includes statements such as “I thought this system was easy to use” and “I felt confident using this system” [46,47]. Scores range from 0 to 100, and an above average score is 68 or higher. Our primary *safety* outcomes were the difference in pain and pain-related fear between baseline and follow-up, as measured by the McGill pain questionnaire-short form (MPQ-sf) [48] and the TSK [49], respectively. Both these constructs are important in chronic pain management, and we wanted to ensure that the GEXP VR application as a potential treatment option did not negatively influence them. The MPQ-sf is a valid measure that asks participants to respond to 15 pain descriptors (eg, throbbing and tender) on a 4-point Likert scale (0 for none to 3 for severe) to measure the person’s pain rating index (PRI) [50]. The MPQ-sf also includes a visual analog scale (VAS) to rate current pain intensity on a continuous scale (0 for no pain to 100 for worst possible pain) and a present pain intensity index, which asks respondents to select 1 of 6 words to describe their pain (0 for no pain to 5 for excruciating pain). We administered the TSK at baseline and follow-up to measure pain-related fear. The TSK is a valid and reliable tool to measure fear of movement in individuals with cLBP [8,41,49]. TSK scores range from 17 to 68, with scores greater than 37 indicating elevated kinesiophobia [8].

### Secondary Measures of Feasibility

To measure pain changes immediately following each module, we asked participants to mark their pain along a VAS before and after each module. VAS measurements are a valid and reliable method to measure pain in individuals with cLBP [51,52]. After each session, participants also filled out a custom-made questionnaire, which included statements such as “I felt challenged,” “It was fun,” and “I felt motivated” on a 0 (not at all) to 10 (extremely) scale (questions included in Multimedia Appendix 1).

### Data Analysis

This study aimed to (1) determine the *GEXP content* validity of the VR application and (2) determine the *feasibility* of individuals with cLBP performing integrated physical activities. We descriptively summarized demographic data.

To examine the GEXP content validity of the graded modules, we used Friedman tests and Dunn post hoc pairwise comparisons with Bonferroni corrections to analyze ordinal data across sessions (avoidance rank and RPE). For continuous data (expected pain and expected concern), we performed a repeated measures analysis of variance (ANOVA) across the VR sessions and performed post hoc pairwise comparisons with Bonferroni corrections. We reported the means and SDs of how participants rated each session’s difficulty compared with the previous sessions.

To evaluate the feasibility of the VR application, we calculated the means and SDs of the TEI-sf, SUS, and postsession questionnaire. We performed paired *t* tests to compare participant pain and pain-related fear from baseline to follow-up. We also used paired *t* tests to examine differences in VAS pain from before to after each module.

We performed Shapiro-Wilk normality tests to confirm the normality of the distribution for dependent variables. For all analyses, we collapsed the VR modules into 3 groups (session 1, session 2, and session 3), based on the 3 VR sessions. We set the alpha level of significance to .05 (two tailed) for all statistical tests. We checked the data for underlying assumptions, and data were described and analyzed using IBM SPSS 25 (IBM Corp).

## Results

### Primary Measures of GEXP Content Validity Results (Hypothesis 1)

Participants assigned higher avoidance, expected pain, and expected concern to the Lucid sessions that are designed to be more challenging (Table 3). A Friedman test revealed that the difference in avoidance was statistically significant (χ^2^_2_=15.1; *P*=.001). Dunn post hoc pairwise comparisons indicated that the difference between session 1 and session 3 was significant after Bonferroni correction (*P*=.002). The avoidance difference between sessions 1 and 2 and the avoidance difference between sessions 2 and 3 were not significant. A repeated measures ANOVA revealed significant differences across the sessions for expected pain (*F*_2,22_=17.9; *P*<.001) and expected concern for harming their back (*F*_2,22_=16.83; *P*<.001). Pairwise comparisons revealed that expected pain was significantly higher for session 3 than for session 1 (*P*=.002) and session 2 (*P*=.03) after Bonferroni correction. The difference in expected pain between session 1 and session 2 was also significant (*P*=.003). Similarly, pairwise comparisons revealed that expected concern was significantly higher for session 3 than for session 1 (*P*=.002) and session 2 (*P*=.008) after Bonferroni correction. The difference in expected concern between session 1 and session 2 was also significant (*P*=.03).

**Table 3:** Avoidance rank, expected pain, and expected concern for harm.

Measures and sessions		Values	95% CI
**Avoidance rank (possible score range 0-6),** **median (IQR)**			
	Session 1	2.5 (1.4)^a^	2.0-2.8
	Session 2	3.3 (1.8)	2.6-3.0
	Session 3	5.5 (0.8)^a^	4.7-5.6
**Expected pain** **(** **possible score range 0-100), mean (SD)**			
	Session 1	38.3 (25.4)^a,b^	22.2-54.5
	Session 2	54.2 (25.2)^b,c^	38.2-70.2
	Session 3	69.4 (22.2)^a,c^	55.3-83.5
**Expected concern (possible score range 0-100), mean (SD)**			
	Session 1	42.3 (27.5)^b,c^	24.8-59.8
	Session 2	57.5 (31.2)^b,c^	37.7-77.3
	Session 3	74.4 (30.9)^a,c^	54.7-94.9

^a^Significant pairwise comparison between sessions 1 and 3.

^b^Significant pairwise comparison between sessions 1 and 2.

^c^Significant pairwise comparison between sessions 2 and 3.

### Secondary Measures of GEXP Content Validity Results

The average RPE slightly increased over the sessions, and 83% (10/12) of the participants reported a higher exertion on session 3 compared with session 1 (Table 4). The Friedman test indicated that the difference in exertion between sessions was significant (χ^2^_2_=10.0; *P*=.007). Post hoc pairwise comparisons using Dunn method indicated that the difference between session 1 and session 3 was significant after Bonferroni correction (*P*=.009). The RPE difference between sessions 1 and 2 and the difference between sessions 2 and 3 were not significant.

**Table 4:** Rating of perceived exertion.

Sessions	Median (IQR)^a^	95% CI
1	14.0 (4.75)^b^	13.2-16.3
2	15.0 (4.75)	14.6-17.5
3	17.0 (4.75)^b^	15.4-18.2

^a^Possible scores from 6 to 20.

^b^Significant pairwise comparison between sessions 1 and 3.

On the difficulty rating questions, participants reported that the session 3 modules were more difficult than the session 1 modules (mean 6.1, SD 3.6) and session 2 modules (mean 5.9, SD 3.5).

### Primary Measures of Feasibility Results (Hypotheses 2 and 3)

#### TEI: Acceptability

Participants responded positively to the VR application as an acceptable potential intervention for cLBP. The average TEI score was 32.5 (SD 4.9), which is above the acceptability cutoff score of 27. Scores ranged from 26 to 41, and 92% (11/12) of participants responded at the cutoff score or above.

#### SUS: Usability

The average SUS score was 76.7 (SD 13.0). Scores ranged from 52.5 to 92.5, and 75% (9/12) of the participants reported that the system was usable.

#### Pain and Pain-Related Fear: Safety

From baseline to follow-up, there were no overall changes in the MPQ-sf PRI (*P*=.20), MPQ-sf VAS (*P*=.73), or TSK (*P*=.58). In total, 67% (8/12) of the participants had improved MPQ-sf PRI scores at follow-up, and participants who improved showed an average decrease of 7.8 (SD 5.1) points on the PRI.

### Secondary Measures of Feasibility Results

In most of the VR modules, there were no statistically significant changes in VAS pain ratings. However, VAS pain significantly increased during module 6 (*P*=.02), the most physically challenging module. In module 6, average post-VR pain rose to 51.5 (SD 32.01) from the 44.0 (SD 28.5) pre-VR pain levels. On the postsession questionnaire, participants responded positively to the modules (Multimedia Appendix 1). After session 3, participants responded that even though the modules were challenging (mean 9.0, SD 1.4), they were also motivating (mean 9.0, SD 1.3), enjoyable (mean 8.7, SD 1.8), and fun (mean 9.1, SD 1.3).

## Discussion

### Principal Findings

In this study, we aimed to (1) determine the GEXP content validity of the VR application and (2) determine the feasibility of individuals with cLBP performing integrated physical activities. Participant responses supported that the VR modules provided progressive exposure to fearfully perceived tasks by assigning greater avoidance to the modules designed to elicit greater fearful appraisals and present greater challenges. In concurrence with the FAM, participants assigned the highest expected pain and concern for harming their back to the modules that they ranked with higher avoidance. For feasibility, the TEI and SUS scores indicated that participants with high fear and cLBP found the VR application an acceptable approach to treat cLBP and usable as a system. Additionally, the VR application was safe, as participants successfully completed the GEXP VR protocol without negative effects on pain or pain-related fear.

The GEXP VR application is an important step in using VR as a potential treatment option because it allowed participants to gradually practice real-life movements through VR activities that require combinations of walking and reaching. Many daily tasks require a person to combine walking and reaching abilities, such as walking to open a door or carrying a bag of groceries. Previous applications have provided exposure to stationary tasks, but by incorporating these tasks into a walking environment, there may be greater potential to translate learning into real-world activities.

The participant feedback we captured is consistent with other studies that have tested VR apps designed for individuals with cLBP. Thomas et al [28] reported the feasibility and safety of a VR dodgeball intervention that encourages participants to use various amounts of lumbar flexion to achieve VR reaching and bending objectives. They reported that their participants were able to complete the VR activities with no adverse events and that their participants responded positively to the VR activity through agreeing with statements such as “The game was fun” and “The game encouraged me to move”. Yelvar et al [53] reported that passively viewing VR walking scenes during physical therapy may improve pain-related outcomes in individuals with cLBP. Additionally, Fowler et al [54] studied the use of VR to gradually expose veterans with chronic pain to progressively more involved movements. They found that gradual VR exposure was feasible but also reported that users rated the activities, designed to progressively deliver more exposure, with similar intensity ratings [54]. Although these applications are useful to train specific movement tasks and support that VR may be an acceptable way to address chronic pain, the VR application described in this study is the first app, to our knowledge, designed specifically to apply VR GEXP in an interactive walking protocol for individuals with cLBP.

Although we intentionally used a GEXP mechanism in the Lucid VR application to decrease the physical limitations caused by activity avoidance, other factors may have helped participants complete the modules. Distraction from pain is a well-studied mechanism commonly used in acute pain [20,55]. We added components in each module to increase movement exposure, such as adding coins to collect or animals to rescue. These novel components may have helped hold the attention of participants and maintained distraction. Many participants reported that they were less aware of their pain and that the modules distracted them from their pain, and this likely contributed to their expressed acceptance of the VR application. Therefore, although VR GEXP may be a targeted way to progressively challenge participants with cLBP, distraction may be an important component to include in future VR applications.

The lack of observed elevations for pain and pain-related fear across the study sessions reflects that individuals with high fear and cLBP were able to successfully complete the challenging activities without adverse consequences in these domains. In line with our long-term intervention goal to reduce deficits in physical ability caused by activity avoidance, participants with cLBP and elevated pain-related fear exhibited their ability to perform functional movement activities such as walking, reaching, carrying, and crouching despite experiencing pain and pain-related fear.

Our study goal was to test the GEXP content and feasibility of the VR application, which we designed to improve the physical abilities of individuals with cLBP by gradually exposing participants to more difficult challenges. Given that chronic pain can significantly interfere with one’s goals and ability to complete everyday activities, interventions that increase physical ability are valuable. This VR application allows individuals not only to interact in an interesting and challenging VR world, but it provided physical challenges that incorporated body transport and reaching movements in a dynamic and motivating environment.

### Limitations

As our study design was to establish the content validity and feasibility of the GEXP app, all participants received the same activities in the same order. The lack of personalization could have limited the GEXP experience for some of our participants as we only provided exposure to these predefined movement combinations. We do not believe this limitation had a significant effect on our study outcomes as participants generally ranked the activities by avoidance in the expected order. In addition, although we used RPE to measure perceived effort, we did not ask participants to specifically rate the difficulty of each module given their back pain. Participants may have assigned greater effort to the higher intensity modules for reasons other than back-related challenges. Participants who walked faster may have had higher exposure as they were able to progress further along the trail in 3 min. Additionally, we only recruited 13 individuals to test the VR app, and this potentially limits our statistical power. To our knowledge, this is the first study to test a VR GEXP walking protocol for individuals with cLBP, and it was essential to determine whether individuals with cLBP could engage with the system and perform the challenges in the novel walking environment before expanding into a larger sample size.

### Future Directions

The next step in this line of research is to explore the efficacy of the VR application on pain-related health outcomes. For this, we would need a larger sample size and a longer study duration. Although this study focused on GEXP content and feasibility, we would also need to explore how the VR exposure training translates to real-world activities by measuring changes in avoidance, disability, and physical function outside of the VR setting. Additionally, future iterations could improve the GEXP experience by allowing the participants to rank a greater number of activities and then experience modules tailored to how they rank their avoidance of each task. This would improve the personalization of the VR application and could allow participants to experience a tailored program more relevant to their daily living. Although our study focused on the general perceived effort of module activities, future studies could also specifically ask about the perceived back-related challenge of activities to better characterize the GEXP. In line with using VR to provide a tailored therapeutic experience, future studies should also explore how age, gender, and pain status influence participation in GEXP VR therapies.

### Conclusions

We have established that the VR modules provided progressive challenges and were feasible for individuals with cLBP and high pain-related fear. The locomotion-enabled VR modules allowed users to freely walk and complete challenging physical activities in a motivating environment that participants thought was acceptable, usable, and safe. Expectation ratings, RPE, and module difficulty responses support that the sessions and comprising modules provided a progressive challenge, in line with GEXP protocols. Despite presenting activities likely for individuals with high fear to avoid, the graded VR walking challenges did not increase pain or fear of movement. The VR modules provided exposure to physical activity challenges that integrate reaching, walking, crouching, and carrying weights while also providing a safe bout of exercise and an enjoyable gaming experience.

## Appendix

Multimedia Appendix 1 Postsession questionnaire and responses.

## Abbreviations

ANOVA: analysis of variance
cLBP: chronic low back pain
FAM: fear-avoidance model
GEXP: graded exposure
HMD: head-mounted display
KA-MX: KineAssist-MX
MPQ-sf: McGill pain questionnaire-short form
PRI: pain rating index
RPE: rating of perceived exertion
SUS: system usability scale
TEI-sf: treatment evaluation inventory-short-form
TSK: Tampa scale for kinesiophobia
VAS: visual analog scale
VR: virtual reality

## References

[1] Rubin DI (2007). Epidemiology and risk factors for spine pain. Neurol Clin.

[2] Centers for Disease Control and Prevention (CDC) (2009). Prevalence and most common causes of disability among adults--United States, 2005. MMWR Morb Mortal Wkly Rep.

[3] Chou R, Qaseem A, Snow V, Casey D, Cross JT, Shekelle P, Owens DK, Clinical Efficacy Assessment Subcommittee of the American College of Physicians, American College of Physicians, American Pain Society Low Back Pain Guidelines Panel (2007). Diagnosis and treatment of low back pain: a joint clinical practice guideline from the American College of Physicians and the American Pain Society. Ann Intern Med.

[4] Engel GL (1977). The need for a new medical model: a challenge for biomedicine. Science.

[5] Gatchel RJ, Peng YB, Peters ML, Fuchs PN, Turk DC (2007). The biopsychosocial approach to chronic pain: scientific advances and future directions. Psychol Bull.

[6] Crombez G, Vlaeyen JW, Heuts PH, Lysens R (1999). Pain-related fear is more disabling than pain itself: evidence on the role of pain-related fear in chronic back pain disability. Pain.

[7] Leeuw M, Goossens ME, Linton SJ, Crombez G, Boersma K, Vlaeyen JW (2007). The fear-avoidance model of musculoskeletal pain: current state of scientific evidence. J Behav Med.

[8] Vlaeyen JW, Kole-Snijders AM, Boeren RG, van Eek H (1995). Fear of movement/(re)injury in chronic low back pain and its relation to behavioral performance. Pain.

[9] Vlaeyen JW, Linton SJ (2000). Fear-avoidance and its consequences in chronic musculoskeletal pain: a state of the art. Pain.

[10] Al-Obaidi SM, Al-Zoabi B, Al-Shuwaie N, Al-Zaabie N, Nelson RM (2003). The influence of pain and pain-related fear and disability beliefs on walking velocity in chronic low back pain. Int J Rehabil Res.

[11] Thomas JS, France CR (2007). Pain-related fear is associated with avoidance of spinal motion during recovery from low back pain. Spine (Phila Pa 1976).

[12] Gardner T, Refshauge K, McAuley J, Goodall S, Hübscher M, Smith L (2015). Patient led goal setting in chronic low back pain-what goals are important to the patient and are they aligned to what we measure?. Patient Educ Couns.

[13] Staal JB, Rainville J, Fritz J, van Mechelen W, Pransky G (2005). Physical exercise interventions to improve disability and return to work in low back pain: current insights and opportunities for improvement. J Occup Rehabil.

[14] Sullivan MD, Ballantyne JC (2016). Must we reduce pain intensity to treat chronic pain?. Pain.

[15] George SZ, Wittmer VT, Fillingim RB, Robinson ME (2010). Comparison of graded exercise and graded exposure clinical outcomes for patients with chronic low back pain. J Orthop Sports Phys Ther.

[16] Trost Z, France CR, Thomas JS (2009). Examination of the photograph series of daily activities (PHODA) scale in chronic low back pain patients with high and low kinesiophobia. Pain.

[17] Leeuw M, Goossens ME, van Breukelen GJ, Boersma K, Vlaeyen JW (2007). Measuring perceived harmfulness of physical activities in patients with chronic low back pain: the photograph series of daily activities--short electronic version. J Pain.

[18] Leeuw M, Goossens ME, van Breukelen GJ, de Jong JR, Heuts PH, Smeets RJ, Köke AJ, Vlaeyen JW (2008). Exposure in vivo versus operant graded activity in chronic low back pain patients: results of a randomized controlled trial. Pain.

[19] Woods MP, Asmundson GJ (2008). Evaluating the efficacy of graded in vivo exposure for the treatment of fear in patients with chronic back pain: a randomized controlled clinical trial. Pain.

[20] Trost Z, Parsons TD (2014). Beyond distraction: virtual reality graded exposure therapy as treatment for pain-related fear and disability in chronic pain. J Appl Biobehav Res.

[21] Jaffe DL, Brown DA, Pierson-Carey CD, Buckley EL, Lew HL (2004). Stepping over obstacles to improve walking in individuals with poststroke hemiplegia. J Rehabil Res Dev.

[22] Levac DE, Huber ME, Sternad D (2019). Learning and transfer of complex motor skills in virtual reality: a perspective review. J Neuroeng Rehabil.

[23] Holden MK (2005). Virtual environments for motor rehabilitation: review. Cyberpsychol Behav.

[24] Lozano-Quilis J, Gil-Gómez H, Gil-Gómez JA, Albiol-Pérez S, Palacios-Navarro G, Fardoun HM, Mashat AS (2014). Virtual rehabilitation for multiple sclerosis using a kinect-based system: randomized controlled trial. JMIR Serious Games.

[25] Trost Z, Zielke M, Guck A, Nowlin L, Zakhidov D, France CR, Keefe F (2015). The promise and challenge of virtual gaming technologies for chronic pain: the case of graded exposure for low back pain. Pain Manag.

[26] Kerr KM (1990). Movement science: foundations for physical therapy in rehabilitation. Physiotherapy.

[27] Adams DL (1999). Develop better motor skill progressions with gentile's taxonomy of tasks. J Phy Educ Recr Dance.

[28] Thomas JS, France CR, Applegate ME, Leitkam ST, Walkowski S (2016). Feasibility and safety of a virtual reality dodgeball intervention for chronic low back pain: a randomized clinical trial. J Pain.

[29] France CR, Thomas JS (2018). Virtual immersive gaming to optimize recovery (VIGOR) in low back pain: a phase II randomized controlled trial. Contemp Clin Trials.

[30] (1998). ISO 9241-11:1998 Ergonomic Requirements for Office Work With Visual Display Terminals (Vdts) — Part 11: Guidance on Usability. ISO - International Organization for Standardization.

[31] Kelley ML, Heffer RW, Gresham FM, Elliott SN (1989). Development of a modified treatment evaluation inventory. J Psychopathol Behav Assess.

[32] Peshkin M (2005). KineAssist: a Robotic Overground Gait and Balance Training Device. Proceedings of the 9th International Conference on Rehabilitation Robotics.

[33] Patton J, Brown DA, Peshkin M, Santos-Munné JJ, Makhlin A, Lewis E, Colgate EJ, Schwandt D (2008). KineAssist: design and development of a robotic overground gait and balance therapy device. Top Stroke Rehabil.

[34] Wang J, Hurt CP, Capo-Lugo CE, Brown DA (2015). Characteristics of horizontal force generation for individuals post-stroke walking against progressive resistive forces. Clin Biomech (Bristol, Avon).

[35] Hurt CP, Wang J, Capo-Lugo CE, Brown DA (2015). Effect of progressive horizontal resistive force on the comfortable walking speed of individuals post-stroke. J Neuroeng Rehabil.

[36] Rumble DD, Hurt CP, Brown DA (2018). Step-by-step variability of swing phase trajectory area during steady state walking at a range of speeds. PLoS One.

[37] Graham SA, Hurt CP, Brown DA (2018). Minimizing postural demands of walking while still emphasizing locomotor force generation for nonimpaired individuals. IEEE Trans Neural Syst Rehabil Eng.

[38] Dionisio VC, Brown DA (2016). Collaborative robotic biomechanical interactions and gait adjustments in young, non-impaired individuals. J Neuroeng Rehabil.

[39] Kugler K, Wijn J, Geilen M, de Jong J, Vlaeyen J (1999). The photograph series of daily activities (PHODA). Pain-Related Fear:Exposure-Based Treatment of Chronic Pain: Exposure-Based Treatment.

[40] Trost Z, France CR, Thomas JS (2008). Exposure to movement in chronic back pain: evidence of successful generalization across a reaching task. Pain.

[41] Roelofs J, Goubert L, Peters ML, Vlaeyen JW, Crombez G (2004). The Tampa Scale for Kinesiophobia: further examination of psychometric properties in patients with chronic low back pain and fibromyalgia. Eur J Pain.

[42] Borg G (1998). Borg's Perceived Exertion and Pain Scales.

[43] Demoulin C, Verbunt JA, Winkens B, Knottnerus JA, Smeets RJ (2010). Usefulness of perceived level of exertion in patients with chronic low back pain attending a physical training programme. Disabil Rehabil.

[44] Amano S, Ludin AF, Clift R, Nakazawa M, Law TD, Rush LJ, Manini TM, Thomas JS, Russ DW, Clark BC (2016). Effectiveness of blood flow restricted exercise compared with standard exercise in patients with recurrent low back pain: study protocol for a randomized controlled trial. Trials.

[45] Penn T, Browning W, France C, Hardee G, Zielke M, Trost Z (2017). Attitudes toward a virtual reality physical activity intervention among veterans with chronic low back pain. J Pain.

[46] Brooke J (2013). SUS: a retrospective. J Usability Stud.

[47] Bangor A, Kortum PT, Miller JT (2008). An empirical evaluation of the system usability scale. Int J Hum-Comput Int.

[48] Melzack R (1987). The short-form McGill pain questionnaire. Pain.

[49] Goubert L, Crombez G, Van Damme S, Vlaeyen JW, Bijttebier P, Roelofs J (2004). Confirmatory factor analysis of the Tampa Scale for Kinesiophobia: invariant two-factor model across low back pain patients and fibromyalgia patients. Clin J Pain.

[50] Wright KD, Asmundson GJ, McCreary DR (2001). Factorial validity of the short-form McGill pain questionnaire (SF-MPQ). Eur J Pain.

[51] Hawker GA, Mian S, Kendzerska T, French M (2011). Measures of adult pain: visual analog scale for pain (VAS Pain), numeric rating scale for pain (NRS Pain), McGill pain questionnaire (MPQ), short-form McGill pain questionnaire (SF-MPQ), chronic pain grade scale (CPGS), short form-36 bodily pain scale (SF-36 BPS), and measure of intermittent and constant osteoarthritis pain (ICOAP). Arthritis Care Res (Hoboken).

[52] Olaogun MO, Adedoyin RA, Ikem IC, Anifaloba OR (2009). Reliability of rating low back pain with a visual analogue scale and a semantic differential scale. Physiother Theor Pr.

[53] Yelvar GD, Çırak Y, Dalkılınç M, Demir Y, Guner Z, Boydak A (2017). Is physiotherapy integrated virtual walking effective on pain, function, and kinesiophobia in patients with non-specific low-back pain? Randomised controlled trial. Eur Spine J.

[54] Fowler CA, Ballistrea LM, Mazzone KE, Martin AM, Kaplan H, Kip KE, Ralston K, Murphy JL, Winkler SL (2019). Virtual reality as a therapy adjunct for fear of movement in veterans with chronic pain: single-arm feasibility study. JMIR Form Res.

[55] Malloy KM, Milling LS (2010). The effectiveness of virtual reality distraction for pain reduction: a systematic review. Clin Psychol Rev.

