# Supplementary material for: A Graded Exposure, Locomotion-Enabled Virtual Reality App During Walking and Reaching for Individuals With Chronic Low Back Pain: Cohort Gaming Design
Source: JMIR Serious Games. 2020 Aug 10;8(3):e17799. doi: 10.2196/17799 (PMC7445609; doi:10.2196/17799)
Supplement: Multimedia Appendix 1 [file games_v8i3e17799_app1.doc]

Appendix 1: Postsession questionnaire and responses.

| **Item** | **Session 1** | **Session 2** | **Session 3** |
| --- | --- | --- | --- |
| 1. It was enjoyable | 8.55 (1.92) | 8.75 (1.42) | 8.67 (1.83) |
| 1. It was boring | 1.64 (1.29) | 2.00 (2.13) | 1.25 (0.754) |
| 1. It was fun | 8.91 (2.12) | 8.17 (2.62) | 9.08 (1.31) |
| 1. It was interesting | 8.91 (2.12) | 7.91 (2.55) | 8.83 (1.40) |
| 1. It was tiring | 7.18 (3.12) | 7.00 (3.28) | 7.67 (2.90) |
| 1. I felt good | 7.82 (2.82) | 7.83 (1.80) | 8.42 (1.73) |
| 1. I felt challenged | 8.40 (2.59) | 7.92 (2.75) | 9.00 (1.41) |
| 1. I felt annoyed | 1.64 (1.50) | 1.67 (1.16) | 1.75 (1.29) |
| 1. I felt motivated | 8.27 (2.76) | 8.42 (1.88) | 9.00 (1.28) |
| 1. I felt comfortable playing the game | 8.55 (1.81) | 8.42 (3.05) | 8.50 (1.98) |
| 1. I wanted to keep playing the game | 6.82 (3.89) | 7.25 (3.05) | 6.67 (3.00) |
| 1. I found the game easy to use | 7.00 (3.23) | 7.50 (2.54) | 7.17 (2.76) |
| 1. I had to put a lot of effort into it | 7.27 (2.69) | 6.83 (3.22) | 7.17 (3.30) |
| 1. I found the game physically demanding | 7.55 (3.30) | 8.08 (2.81) | 7.25 (3.84) |
| 1. I felt immersed in the game | 7.73 (2.49) | 6.25 (3.55) | 6.33 (3.39) |
| 1. I was less aware of my pain during gameplay | 6.82 (2.96) | 7.00 (2.63) | 6.67 (3.17) |
| 1. I was more aware of my pain during gameplay | 5.09 (3.62) | 5.08 (3.63) | - 1. (3.53) |
| 18A. Did you experience pain? | No: 11  Yes: 1 | No: 10  Yes: 2 | No: 10  Yes: 2 |
| 18B. If yes, please describe. | “Lower back” | “I got tired fast”  “Legs and lower back” | “I was already hurting and noticed it when I walked”  “Lower back” |
| 1. How difficult was it to learn how to do these activities? | 1.45 (1.13) | 3.64 (4.03) | 3.50 (3.29) |
| 1. To what extent did doing this task distract you from your pain? | 6.36 (3.20) | 6.45 (2.81) | 7.83 (2.04) |
| 1. Compared to the activities at your last visit, how much more difficult were today’s activities? |  |  | 5.92 (3.53) |
| 1. Compared to the activities at your first visit, how much more difficult were today’s activities? |  |  | 6.08 (3.58) |
